# Evaluation of physiological and biochemical responses in different seasons in Surti buffaloes

**DOI:** 10.14202/vetworld.2015.727-731

**Published:** 2015-06-17

**Authors:** Sandhya S. Chaudhary, Virendra Kumar Singh, Ramesh C. Upadhyay, Gopal Puri, Arjun B. Odedara, Pankaj A. Patel

**Affiliations:** 1 Department of Veterinary Physiology and Biochemistry, Vanbandhu College of Veterinary Science and Animal Husbandry, Navsari Agricultural Univesrity, Navsari - 396 450, Gujarat, India; 2 Division of Dairy Cattle Physiology, National Dairy Research Institute, Karnal - 132 001, Haryana, India

**Keywords:** biochemical, heat stress, physiological, surti buffalo, temperature humidity index

## Abstract

**Aim:**

This study was conducted to evaluate the impact of hot dry, hot humid and comfortable season on physiological, hematological, biochemical, and oxidative stress parameters in Surti buffaloes.

**Materials and Methods:**

Ten lactating Surti buffaloes of similar physiological status were selected. Based on the temperature-humidity index (THI), their natural exposure to the environment was categorized as hot dry (THI_1_), hot humid (THI_2_) and moderate winter/comfort season (THI_3_). Blood/serum samples were collected and analyzed for physiological, hematological, biochemical, and oxidative stress parameters. The results were analyzed using standard statistical methods.

**Results:**

With increase in THI, significant rise in physiological parameters such as respiration rate (RR), hematological parameters such as red blood cell (RBC), hematocrit, hemoglobin (Hb) and mean cell Hb concentration (MCHC), biochemical parameters such as alanine aminotransferase (ALT), Na, K, creatinine, blood urea nitrogen, Mn, Cu and Zn, hormones such as cortisol and oxidative stress parameters such as glutathione peroxidase (GPx), superoxide dismutase (SOD), lipid peroxide (LPO) and total antioxidant status (TAS) and significant decline in glucose, cholesterol and triiodothyronine (T_3_) was observed.

**Conclusion:**

It was concluded that THI is a sensitive indicator of heat stress and is impacted by ambient temperature more than the relative humidity in buffaloes. Higher THI is associated with significantly increased RR, total RBC count, Hb, hematocrit, MCHC, ALT, urea, sodium, creatinine, triiodothyronine, SOD, GPx, LPO and TAS and with significant decrease in glucose, cholesterol and triiodothyronine (T_3_).

## Introduction

The buffaloes are essentially shade and water loving animals and are well suited to hot and humid climate, but they exhibit signs of heat stress when exposed to direct solar radiation. This is due to the fact that buffalo’s body absorbs a great deal of solar radiation because of their dark skin and sparse coat or hair, and in addition to that they possess a less efficient evaporative cooling system due to their rather poor sweating ability. Particularly, exposure of buffaloes to the latter conditions evokes a series of drastic changes in biological functions that include depression in feed intake, efficiency and utilization [1], disturbances in metabolism of water, protein, energy and mineral balances, enzymatic reactions, hormonal secretions and blood metabolites [2]. Such changes result in impairment of growth, production, and reproduction performance.

The effect of heat stress is aggravated when heat stress is accompanied by high ambient humidity. Temperature humidity indices (THI) are used to know the amount of heat stress in animals [3]. It takes into account the temperature and the humidity of the environment to which the animals are exposed.

Most of the studies in relation to heat stress have been done on exposure of buffaloes to direct solar radiation. However there are very few studies conducted on buffaloes related to microenvironment to which animals are confined. Therefore, the present study was conducted on Surti buffaloes in the microenvironment of three different seasons along with THI with the objective to find out the markers of heat stress.

## Materials and Methods

### Ethical approval

The experiment was part of NICRA project (ICAR-NDRI) and it followed the guidelines of Institutional Animal Ethics Committee.

### Animal selection

This study was conducted on 10 lactating Surti buffaloes in same stage (4-5^th^ lactation) of lactation maintained at Livestock Research Station, NAU, Navsari after successive natural exposure of the same animals to hot dry, hot humid and moderate winter (comfortable season). These animals were fed as per ICAR 1998 feeding standards.

### Meteorological observations

Data of temperature and humidity of the shed in which animals were kept were recorded with the help of data logger for last 15 days. THI was calculated from mean temperature and mean relative humidity and using the formula of Mader *et al*. [4].

### Sample collection and analysis

Blood sample in K_3_ ethylenediaminetetraacetic acid and serum collection tubes were carried out in hot dry (THI_1_), hot humid (THI_2_) and moderate winter/comfort season (THI_3_). Blood samples were analyzed by hematology analyzer (Medonic CA620 VET - Boule Medical AB) for hematological parameters whereas the serum was separated by centrifugation at 3000 rpm for 10 min and stored till analyzed. The serum was analyzed for total protein, cholesterol, glucose, chloride, calcium, magnesium, alanine aminotransferase (ALT) (U/L), AST (U/L), non-esterified fatty acid (mmol/L), creatinine (mg/dl) and blood urea nitrogen (BUN) (mg/dl) using “Randox” kits (Randox Laboratories Limited, United Kingdom). Triiodothyroxine (T_3_), thyroxine (T_4_) and cortisol were measured by standard enzyme-linked immunosorbent assay technique using assay kit (Labor Diagnostica Nord GmbH & Co. KG, Nordhorn). Sodium and potassium were analyzed by systronic make 128 flame photometer. Iron-Fe^+2^ (ppm), manganese-Mn^+2^ (ppm), copper-Cu^+2^ (ppm) and zinc-Zn^+2^ (ppm) were analyzed on ECIL make atomic absorption spectrophotometer. Oxidative stress parameters *viz*. glutathione peroxidase (GPx) (UV method of Paglia and Valentine [5]), superoxide dismutase (SOD) [6] and lipid peroxides (LPO) in terms of malondialdehyde (MDA) production [7]. Red blood cell (RBC) hemolysate was used for analysis of oxidative stress parameters. Total antioxidant status (TAS) was estimated [8] using “Randox” kits (Randox Laboratories Limited, United Kingdom).

### Statistical analysis

The data were subjected to analysis of variance [9] and the means were tested for significance by Duncan’s multiple range test [10].

## Results

The ambient temperature and relative humidity for hot dry (THI_1_), hot humid (THI_2_) and moderate winter/comfort season (THI_3_) was 31.40°C, 60.30%; 28.10°C, 85.60% and 22.10°C, 61.00% respectively. Based on this data results of THI calculated were as follows: THI_1_=81.7 for hot dry, THI_2_=80.6 for hot humid and THI_3_=68.72 for comfort season.

Marked increase in respiration rate (RR) occurred with the increase in THI. RBC, hematocrit, hemoglobin (Hb) and mean cell Hb concentration (MCHC) increased significantly with an increase in THI while the rest of the hematological parameters was found to be non-significant (Table-1). Table-2 shows the changes in biochemical parameters at three different THI. With the increase in THI, a significant increase in ALT, Na, K, creatinine, BUN and trace elements such as Mn, Cu and Zn and a significant decrease in glucose and cholesterol was observed. Among the hormones studied there was a significant decrease in T_3_ with an increase in THI while the cortisol concentration significantly increased at THI_1_ as compared to THI_3_ (Table-2). Mean±SE values of the oxidative stress parameters are given in Table-3. The oxidative stress parameters *viz*. GPx, SOD, LPO and TAS increased significantly with an increase in THI.

**Table-1:** Physiological and hematological parameters (mean±SE) in different seasons.

Parameters	Hot dry (THI_1_-81.7)	Hot humid (THI_2_-80.6)	Comfort (THI_3_-68.72)
Rectal temperature (°F)	100.23±0.27	100.19±0.19	99.35±0.41
RR (breaths/min)	38.40±1.76**	24.3±0.70**	22.7±0.60**
Heart rate (beats/min)	70.2±1.53	68.8±1.34	69.3±1.81
Total RBC count (millions/µl)	7.03±0.33**	5.99±0.24**	5.22±0.47**
MCV (μm^3^)	46.38±1.43	46.8±1.17	45.7±1.64
HCT (%)	32.34±1.06**	27.8±1.34**	23.98±2.41**
Total platelet count (×10^3^/µl)	248.2±16.61	237.9±15.6	220.6±10.96
Total WBC count (×10^3^/µl)	15.28±1.72	13.06±1.67	15.16±1.62
Hb (g/dl)	11.36±0.36*	10.29±0.48*	8.79±0.82*
MCH (pg)	16.29±0.46	17.21±0.45	16.86±0.52
MCHC (g/dl)	35.19±0.34**	36.82±0.34**	36.96±0.42**
LYM (%)	49.7±4.40	50.45±3.88	43.38±4.93
MID cell (%)	7.93±0.54	8.2±0.67	7.55±0.32
GRAN (%)	42.37±4.10	41.15±3.32	49.07±4.71

* Significance at p≤0.05;

** Significance at p≤0.01,

SE=Standard deviation, RR=Respiration rate, WBC=White blood cell, MCV=Mean cell volume, Hb=Hemoglobin, MCH=Mean cell hemoglobin, MCHC=Mean cell hemoglobin concentration, LYM=Lymphocytes, MID=Midsized cell, GRAN=Granulocytes

**Table-2:** Biochemical parameters (mean±SE) in different seasons.

Parameters	Hot dry (THI_1_-81.7)	Hot humid (THI_2_-80.6)	Comfort (THI_3_-68.72)
AST (U/L)	119.9±4.03	131±7.91	121.12±5.51
ALT (U/L)	58.08±3.02**	55.46±5.00**	39.67±4.53**
Total protein (g/dl)	6.67±0.48	7.24±0.19	6.35±0.21
Albumin (g/dl)	3.78±0.10	3.75±0.12	4.11±0.16
Cholesterol (mg/dl)	103.4±10.72**	75.5±8.50**	118.6±11.75**
Glucose (mg/dl)	47.84±1.38**	64.6±3.22**	73.1±3.2**
Sodium-Na (mmol/L)	149.50±3.11**	135.19±5.39**	110.14±4.33**
Potassium-K (mmol/L)	5.25±0.14**	5.616±0.47**	3.82±0.26**
Non esterified fatty acid (mmol/L)	0.44±0.01	0.607±0.24	0.235±0.01
Creatinine (mg/dl)	2.43±0.18**	0.42±0.03**	1.61±0.10**
BUN (mg/dl)	35.00±0.70**	27.99±0.43**	14.8±0.88**
Iron-Fe^+2^ (ppm)	1.40±0.07	1.34±0.1	1.30±0.04
Manganese-Mn^+2^ (ppm)	0.471±0.01**	0.47±0.01**	0.34±0.02**
Copper-Cu^+2^ (ppm)	0.111±0.01**	0.115±0.01**	0.214±0.01**
Zinc-Zn^+2^ (ppm)	0.90±0.13*	0.69±0.07*	0.53±0.04*
Triiodothyronine-T_3_ (ng/ml)	1.88±0.05*	1.79±0.06*	1.66±0.06*
Tetraiodothyroxine-T_4_ (µg/dl)	4.76±0.20	4.69±0.17	4.90±0.27
Cortisol (µg/dl)	1.82±0.10	1.71±0.09	1.55±0.10

* Significance at p≤0.05;

** Significance at p≤0.01,

SE=Standard deviation, AST=Aspartate aminotransferase, ALT=Alanine aminotransferase, NEFA=Non esterified fatty acid, BUN=Blood urea nitrogen

**Table-3:** Oxidative stress parameters (mean±SE) in different seasons.

Parameters	Hot dry (THI_1_-81.7)	Hot humid (THI_2_-80.6)	Comfort (THI_3_-68.72)
GPx (U/ml)	97.24±4.31**	70.97±3.47**	43.42±6.17**
LPO (nmol of MDA/ml of packed cells)	4.83±0.33**	3.57±0.13**	3.16±0.32**
SOD (U)	6.40±0.20**	3.72±0.18**	3.36±0.43**
TAS (mmol/L)	5.28±0.06**	2.92±0.14**	2.01±0.10**

*Significance at p≤0.05;

** Significance at p≤0.01,

SE=Standard deviation, GPx=Glutathione peroxidase, LPO=Lipid peroxide, SOD=Superoxide dismutase, TAS=Total antioxidant status

## Discussion

Even though relative humidity was very high in hot humid season (THI_2_) as compared to that in hot dry season, the slight increase in ambient temperature during hot dry (THI_1_) season has increased THI of hot dry season and it was slightly higher than that of hot humid (THI_2_) season. Thus, it was deduced that as compared to the even major change in relative humidity, a minor change in ambient temperature causes more alteration in THI.

There was a significant increase in RR with an increase in ambient temperature (THI_1_) and the relative humidity (THI_2_) in hot dry season and hot humid season, respectively. Increase in RR indicates that the animals were in heat stress and animals enhance their RR to facilitate heat loss during heat stress and therefore it is recommended as a parameter to assess heat stress along with THI [11]. Scharf *et al*. [12] reported a significant daily increase of 15.4 breaths/min from 600 to 1300 h in *Bos taurus* during heat stress and Wankar *et al*. [13] have also noticed in adult buffaloes significantly increased RR at 35°C and 40°C as compared to 25°C and 30°C. An increase in RR during afternoon as compared to morning as well as during summer (hot dry) as compared to hot humid season in growing and adult Sahiwal cattle have been reported by Chandrabhan *et al*. [14]. Non-significant difference in RT temperature may be due to increasing in RR, which may prevent the increase in rectal temperature in cows [15]. Non-significant difference in heart rate was observed in this study, which is in agreement with the findings of Singh and Bhattacharyya [16].

A significant increase in RBC, Hb, hematocrit (HCT) and MCHC was observed in hot dry as compared to the hot humid season. Increase in RBC (consequent to higher HCT) and Hb may be due to hemoconcentration in the hot dry season. Similar findings of increase in RBC at the beginning of the hot period has been reported by Koubkova *et al*. [17] and Omran *et al*. [18] and in summer season by Toharmat *et al*. [19]. Higher MCH and MCHC during summer have also been reported by Kumar and Pauchaura [20].

Significant decrease in glucose and cholesterol was observed in hot dry and hot humid season as compared to comfortable season. Decrease in glucose [17,21] and cholesterol [12,15] may be due to decrease in feed intake. While a significant increase in ALT and urea was observed in hot dry season. Increase in ALT in hot period is in agreement with the findings of Koubkova *et al*. [17], Chandrabhan *et al*. [14] and Singh and Bhattacharyya [16]. The increase in ALT may be due to increase in gluconeogenesis [17,22] or due to some deleterious effect of heat stress on liver activity [23-25]. Increase in urea in the hot dry and hot humid season may be due to catabolism of protein to maintain the metabolic needs of the body. Increase in urea is also an indication of dehydration as reported by Scharf *et al*. [12]. Increase in urea in summer or the hot period has been reported by Koubkova *et al*. [17] and Das *et al*. [26].

Increase in sodium concentration may be due to dehydration. However, it also depends on the amount of water intake which was not measured in the present study and animals had *ad libitum* access to water. The rate of excretion of creatinine is influenced by renal perfusion and glomerular filtration rate. A reduction in blood flow to the kidneys during heat stress may increase the creatinine concentration. Ronchi *et al*. [27,28] recorded decrease in glucose as well as an increase in urea and creatinine as a result of muscular catabolism for energy supply.

In the present study significant increase in T_3_ but non-significant decrease in T_4_ in hot dry period as compared to hot humid period was observed. Decrease in thyroid hormones occurs during heat acclimation and those mammals acclimated to warmer climate adopt this pattern [29,30].

A significant increase in SOD, GPx, LPO and TAS was observed which indicates increased production of the free radical in hot dry, as well as hot humid season. However, the values of the antioxidant parameters in the hot dry season was significantly higher than the hot humid season indicating that hot dry season is more stressful to Surti buffaloes and leads to increased production of free radicals. Higher levels of SOD and GPx in summer season in Murrah buffalo calves, heifers and lactating buffaloes have been reported by Lallawmkimi [31]. LPO in the form of MDA production was found to be higher in the summer season. Thiobarbituric acid reactive substances is also an indicator of LPO which was also found to increase in summer season in cows as observed by Bernabucci *et al*. [30] and also on exposure of animals to sun as compared to animals kept in shade [32]. Quantification of single antioxidant status tells us little about the whole body defense and a number of antioxidants need to be estimated. Therefore, nowadays TAS or total antioxidant status is measured [33]. In the present study the TAS significantly increased in hot dry season but to come to a concluding point still research needs to be conducted.

## Conclusion

From the present study, it can be concluded that THI is a sensitive indicator of heat stress and is impacted by ambient temperature more than the relative humidity in buffaloes. Higher THI is associated with significantly increased RR, total RBC count, Hb, HCT, MCHC, ALT, urea, sodium, creatinine, triiodothyronine, SOD, GPx, LPO and TAS and with significant decrease in glucose and cholesterol. These effects may be direct or indirect by altered feeding pattern, dehydration, etc. which necessitates further studies to ascertain precisely.

## Authors’ Contributions

SSC planned and executed the experiment and prepared the manuscript. RCU was PI of NICRA project (ICAR) and guided for the study. VKS helped in the preparation of the manuscript and along with PAP and ABO conducted blood biochemical analysis. GP helped in blood collection and recording of physiological parameters All authors read and approved the final manuscript.
